# Investigation on the Differences in the Yield, Quality, and Antioxidant Activity of *Camellia vietnamensis* Seed Oil Between the Fallen Fruits Caused by Typhoons and the Normally Harvested Fruits

**DOI:** 10.3390/molecules31111812

**Published:** 2026-05-25

**Authors:** Chenyu Jiang, Muhammad Sajjad, Kaibing Zhou

**Affiliations:** 1 School of Breeding and Multiplication (Sanya Institute of Breeding and Multiplication), Hainan University, Sanya 572025, China; 23220951310128@hainanu.edu.cn (C.J.); drmuhammadsajjad@hainanu.edu.cn (M.S.); 2 Key Laboratory of Quality Regulation of Tropical Horticultural Crop in Hainan Province, School of Tropical Agriculture and Forestry, Hainan University, Haikou 570228, China; 3 Yunnan International Joint Laboratory of Durian Functional Genomics, College of Landscape and Horticulture, Yunnan Agricultural University, Kunming 650201, China; 4 Key Laboratory of Vegetable Biology of Yunnan Province, College of Landscape and Horticulture, Yunnan Agricultural University, Kunming 650201, China

**Keywords:** typhoons, *Camellia vietnamensis*, yield of seed oil, quality of seed oil, antioxidant activity, volatile compounds

## Abstract

The fallen *Camellia vietnamensis* fruits caused by typhoons are usually collected by the farmers to be processed into oil in order to decrease the loss of the disaster. Then, this report investigates the difference in the yield, quality, and antioxidant activity of the seed oil between the fallen fruits caused by the typhoons and the normally harvested fruits. The yield of seed oil from fallen fruits caused by typhoons (HCA) was significantly lower than that of normally harvested fruits (HCB). The physicochemical properties of HCA showed signs of quality deterioration. HCA seemed to optimize the fatty acid composition. HCA exhibited stronger DPPH^·^ radical scavenging, ABTS^·+^ inhibitory, and ferric ion-reducing activities. Thirty-four volatile compounds were identified in both samples. HCA showed higher levels of antioxidant-rich volatiles. Overall, this investigation demonstrates that the fallen fruits caused by typhoons lead to significant seed oil yield losses and measurable quality deterioration, thereby offering clear, evidence-based insights to support more effective typhoon disaster mitigation strategies.

## 1. Introduction

Oil-tea camellia, belonging to the *Camellia* genus of the Theaceae family, is known as one of the world’s four major woody oil crops, together with olive, palm, and coconut [1]. In recent years, oil-tea camellia seed oil has been widely recognized by the public for its richness in unsaturated fatty acids beneficial to the human body. Compared with the well-known olive oil, oil-tea camellia seed oil generally has a higher content of oleic acid, lower saturated fatty acids, and stronger antioxidant capacity [2]. Oil-tea camellia seed oil is rich in bioactive substances such as tocopherols, sterols, squalene, and polyphenolic compounds [3]. Studies have shown that the active components with antioxidant and cytotoxic properties in oil-tea camellia seed oil determine its anti-inflammatory, antibacterial, and antitumor effects [4]. Therefore, oil-tea camellia seed oil has good application prospects in both edible and other fields such as medicine, chemical industry, and cosmetics [5]. *C. vietnamensis* is a traditional and economically important oil-tea camellia species cultivated in South China. Its seed oil is distinguished by a characteristic fragrance, making it a unique and valuable species within the oil-tea camellia industry [6]. In view of its exceptionally high economic value, horticultural cultivation practices are currently being widely adopted in the oil-tea camellia industry to enhance the yield of oil-tea camellia seed oil [7].

A storm is a disastrous weather system with strong suddenness and great destructiveness, forming a strong tropical cyclone over a vast sea surface above 26 °C in the tropics or subtropics of the Asia-Pacific region. According to the classification of the World Meteorological Organization, such cyclones in the Northwestern Pacific Ocean are called typhoons [8]. In September 2024, super typhoon Category 17 “Capricorn” landed in the north-west of the Hainan islands of China, and it suffered huge economic losses in the agricultural industry after the passage of typhoons [9]. It is particularly important to formulate reasonable and effective post-disaster recovery measures in agricultural and forestry production.

Regarding the forest of *C. vietnamensis*, some trees fell, and a large quantity of leaves and fruits fell off too. After typhoons, the fallen fruits should be collected promptly to process oil because they can bring some economic benefits. However, the changes in the main quality, antioxidant activity and volatile compounds of the oil from the fallen fruits of oil-tea camellia still remain unknown. Thus, it is worth researching, and the results can be used for the evaluation of the yield loss and the changes in the integrated quality of the oil.

Current studies on the impact of typhoons on the oil-tea camellia industry largely emphasize yield losses and post-disaster response measures, whereas proactive, pre-event prevention strategies remain underexplored and are often only considered following initial typhoon impacts. But there was no relevant report on the difference in the main quality, antioxidant activity, and volatile compounds between the oil-tea camellia, including *C. vietnamensis* seed oil from the fallen fruits caused by the meteorological disasters, such as super typhoons or severe typhoons. Therefore, in order to evaluate the damage of the severe typhoons more comprehensively, the objective of this investigation was to understand the differences in *C. vietnamensis* seed oil between the fallen fruits caused by typhoons and the normal harvested fruits, as well as its preliminary chemical mechanism through the identification of fatty acid composition, antioxidant activity, and analysis of bioactive components of oil-tea camellia seed oil.

## 2. Results

### 2.1. Comparison of Yield Traits

The differences in the oil yield traits between HCA and HCB were extremely significant (Table 1). Fresh seed yield rate of fruits, dry kernel yield rate of fruits, oil yield rates of dry seeds and dry kernels, and fruit oil yield rate of HCB were all significantly higher than those of HCA. Except for the fresh seed yield rate of fruits, among all other indicators, the values of HCA decreased by nearly 50% or even more compared with those of HCB. Specifically, the oil yield rate of dry seeds and the oil content rate of fruits decreased by 74.33% and 73.26%, respectively. It indicated that the fallen fruits caused by typhoons caused huge yield losses of *C*. *vietnamensis* seed oil.

### 2.2. Detection and Comparison of Physicochemical Properties

The differences in physicochemical properties of *C*. *vietnamensis* seed oil between HCA and HCB were significant (Table 2). HCA affected the peroxide value, acid value, and saponification value of *C*. *vietnamensis* seed oil. For the peroxide value, HCA was considerably higher than HCB, suggesting that typhoon damage would weaken the antioxidant capacity and storage resistance of *C*. *vietnamensis* seed oil. There was no significant difference in iodine value between HCA and HCB, indicating that typhoon damage had no significant effect on the degree of unsaturation of fatty acids in *C*. *vietnamensis* seed oil. HCA was extremely significantly higher than HCB in the acid value; the acid value of HCA was 208.50% higher than that of HCB. It indicated that the content of free fatty acids in *C*. *vietnamensis* seed oil from HCA increased, thereby accelerating the rancidity of *C*. *vietnamensis* seed oil and damaging its storability, making it difficult to store. For the saponification value, HCB was extremely significantly higher than HCA, indicating that the relative molecular mass of *C*. *vietnamensis* seed oil from HCA was higher, making it less likely to be absorbed by the human body. In summary, typhoons cause fruits to fall, causing the quality deterioration of *C*. *vietnamensis* seed oil.

### 2.3. Determination and Analysis of Fatty Acid Composition

The differences in fatty acid contents of *C*. *vietnamensis* seed oil between HCA and HCB are shown (Table 3). The same 13 kinds of fatty acids were identified in *C*. *vietnamensis* seed oil from HCA and HCB, and among them, the fatty acids were classified into three groups. The saturated fatty acids (SFA) included six kinds, and palmitic acid had the highest relative content, while stearic acid and myristic acid had lower relative concentrations. The monounsaturated fatty acids (MUFA) included five kinds, and oleic acid was dominant, with the content exceeding 75%. The polyunsaturated (PUFA) included two kinds, and linoleic acid was dominant. The ratios of PUFA/SFA of HCA and HCB were 0.65 and 0.63 respectively, and the ratios of MUFA/SFA of HCA and HCB were 5.74 and 5.69 respectively. The ratios of (MUFA + PUFA)/SFA of HCA and HCB were 6.39 and 6.32 respectively, and it seemed to indicate that the oil from HCA was easier to oxidize and go rancid; thus, it basically agreed with the results of the peroxide value and acid value mentioned above. Meanwhile, it seemed to indicate that the oil from HCA, at least, was not harmful to human health. The contents of myristic acid, palmitoleic acid, oleic acid, and α-linolenic acid in HCA were significantly higher, while the contents of stearic acid and nervonic acid were substantially lower, with no significant differences in the contents of other fatty acids. Notably, the changes in fatty acid contents of HCA showed a not-too-obvious trend of favorable quality changes.

### 2.4. Detection and Comparison of Antioxidant Activity

#### 2.4.1. DPPH^·^Free Radical Scavenging Activity

**Figure 1:**
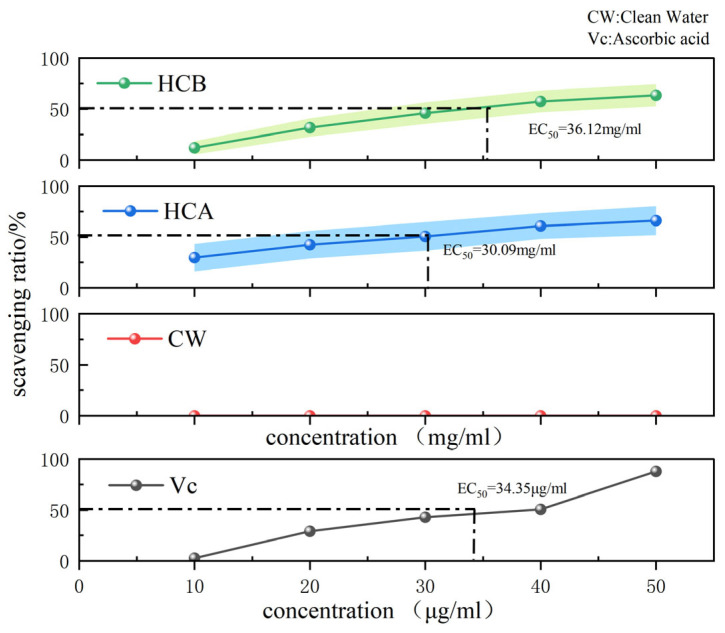
Comparison of the DPPH^·^ scavenging rate of *C. vietnamensis* seed oil between HCA and HCB. Note: HCA symbolizes the typhoon-damaged fallen fruits, and HCB symbolizes the normally harvested fruits.

The DPPH^·^ radical scavenging activity of *C*. *vietnamensis* seed oil from HCA and HCB is presented in Figure 1 and Table 4. On the one hand, the scavenging effect of DPPH^·^ radicals became more significant with the increase in concentration for both. The scavenging rate of DPPH^·^ showed an extremely substantial univariate linear regression relationship with the mass concentration of *C*. *vietnamensis* seed oil, which was positively correlated, indicating that HCA and HCB all had DPPH^·^ radical scavenging activity. On the other hand, both the DPPH^·^ radical scavenging rate and EC_50_ value showed that the DPPH^·^ radical scavenging activity of HCA was stronger than that of HCB, indicating that the seed oil from the fallen fruits caused by typhoons would enhance the DPPH^·^ radical scavenging activity.

#### 2.4.2. ABTS^·+^ Free Radical Inhibitory Activity

**Figure 2:**
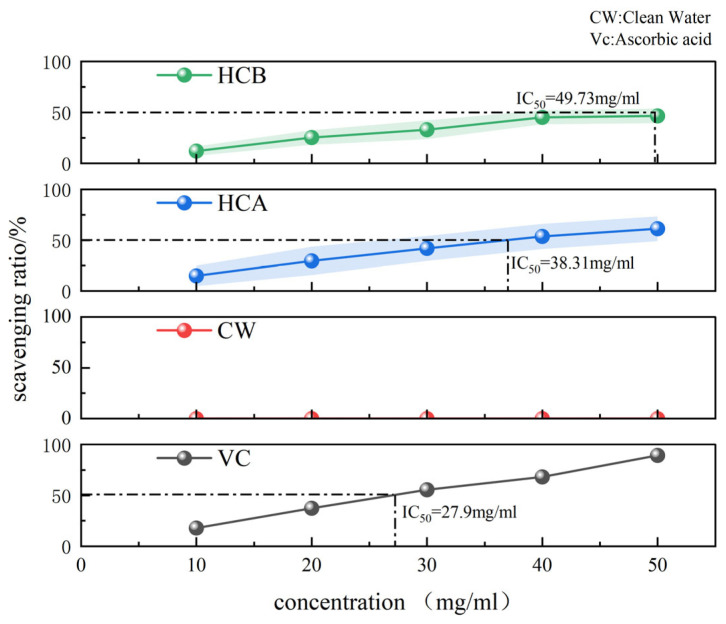
Comparison of the ABTS^·+^ inhibitory rate of *C. vietnamensis* seed oil between HCA and HCB. Note: HCA symbolizes the typhoon-damaged fallen fruits, and HCB symbolizes the normally harvested fruits.

The ABTS^·+^ free radical inhibitory activity of *C*. *vietnamensis* seed oil from HCA and HCB is presented in Figure 2 and Table 5. On the one hand, the inhibitory effect of ABTS^·+^ free radicals became more significant with the increase in concentration for both. The inhibitory rate of ABTS^·+^ free radicals showed an extremely substantial univariate linear regression relationship with the mass concentration of *C*. *vietnamensis* seed oil, which was positively correlated, indicating that HCA and HCB all have ABTS^·+^ free radicals inhibitory activity. On the other hand, both the ABTS^·+^ free radicals inhibitory rate and IC_50_ value showed that the ABTS^·+^ free radicals inhibitory activity of HCA was stronger than that of HCB, indicating that the seed oil from the fallen fruits caused by typhoons would enhance the ABTS^·+^ free radicals inhibitory activity.

#### 2.4.3. Antioxidant Activity Determination by FRAP Assay

**Figure 3:**
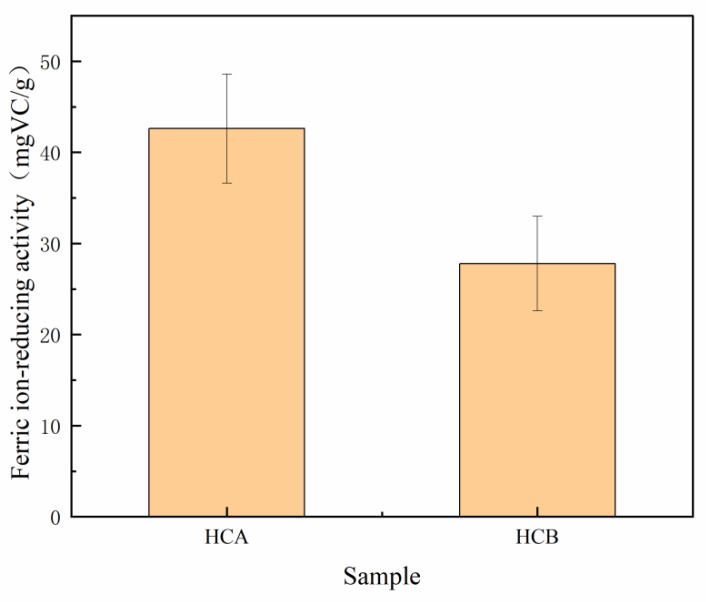
Comparison of the ferric ion-reducing activity by FRAP assay of *C. vietnamensis* seed oil between HCA and HCB. Note: HCA symbolizes the typhoon-damaged fallen fruits, and HCB symbolizes the normally harvested fruits.

The ferric ion-reducing activity by FRAP assay of *C*. *vietnamensis* seed oil from HCA and HCB is presented in Figure 3. Obviously, HCA was extremely significantly higher than HCB. The ferric ion-reducing activity of HCA was 53.31% higher than that of HCB. It indicated that the seed oil from the fallen fruits caused by typhoons would enhance the FRAP antioxidant activity.

#### 2.4.4. Identification and Comparison of Volatile Substances

GC-MS analysis of *C*. *vietnamensis* seed oils from HCA and HCB detected a total of 34 volatile components belonging to seven categories, including one ketone, two alcohols, two organic peroxides, thirteen esters, two terpenes, eight hydrocarbons (seven alkanes and one olefin), one phenol, and five other compounds. The specific compositional differences between HCA and HCB could be seen in Table 6.

The contents of ester compounds (such as Dibutyl phthalate, 11-Octadecenoic acid, methyl ester, etc.) in HCA were the highest, and olefin compounds were not detected. In the *C*. *vietnamensis* seed oil from HCB, ester compounds (such as Dibutyl phthalate, 11-Octadecenoic acid, methyl ester, etc.) had the highest content, while the contents of alkane compounds were the lowest. The seed oil from the fallen fruits caused by typhoons could lead to the disappearance or new formation of certain compounds.

A total of 28 compounds were detected in HCA, and 22 compounds in HCB. Among them, 1,5-Heptadien-4-one, 3,3,6-trimethyl- had the highest content, while Cyclopentanol, dodecamethyl- had the lowest content. Typhoon damage led to increased contents of ketones, alcohols, organic peroxides, partial esters (such as oxalic acid, cyclohexyl octyl ester, glycidyl oleate), olefins, phenols, and some other compounds. It also caused decreased or even disappeared contents of partial esters (such as oxalic acid, cyclohexyl butyl ester, Dibutyl phthalate), terpenes, alkanes, and other compounds. These changes in secondary metabolites reflect the physiological and biochemical changes occurring in *C*. *vietnamensis* fruits and seeds during growth and development, thereby causing variations in the antioxidant activities of *C*. *vietnamensis* seed oil.

### 2.5. PCA

In the PCA plot, samples located closer together indicate smaller compositional differences, whereas samples positioned farther apart indicate greater compositional differences.

**Figure 4:**
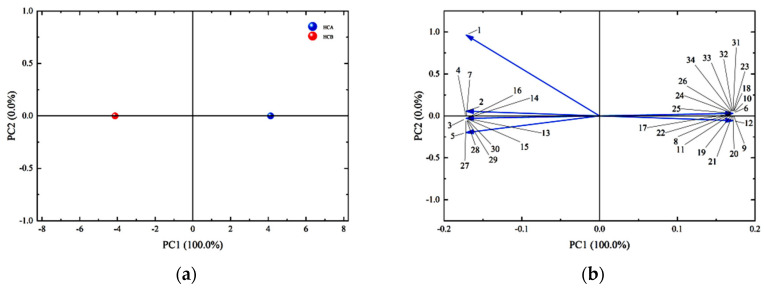
Principal component analysis (PCA) plots of volatile compounds. Note: Numbers 1–34 in the figure above represent volatile substances 1–34 in Table 7. (**a**) Score map, (**b**) correlation loading plot.

The differences in volatile compounds of the seed oil from HCA differed from those from HCB, as shown in the PCA plot (Figure 4), indicating that the composition of volatile compounds and antioxidant activities of the seed oil from HCA significantly differed from those of HCB. HCA, located on the positive axis, was enriched with components with antioxidant activities, such as squalene and cyclononasiloxane, octadecamethyl-, while HCB, located on the negative axis, was enriched with compounds without antioxidant activities. It was agreed with the results mentioned above, such as the difference in the antioxidant activity and the bioactive compounds composition.

## 3. Materials and Methods

### 3.1. Plant Material and Experiment Design

**Figure 5:**
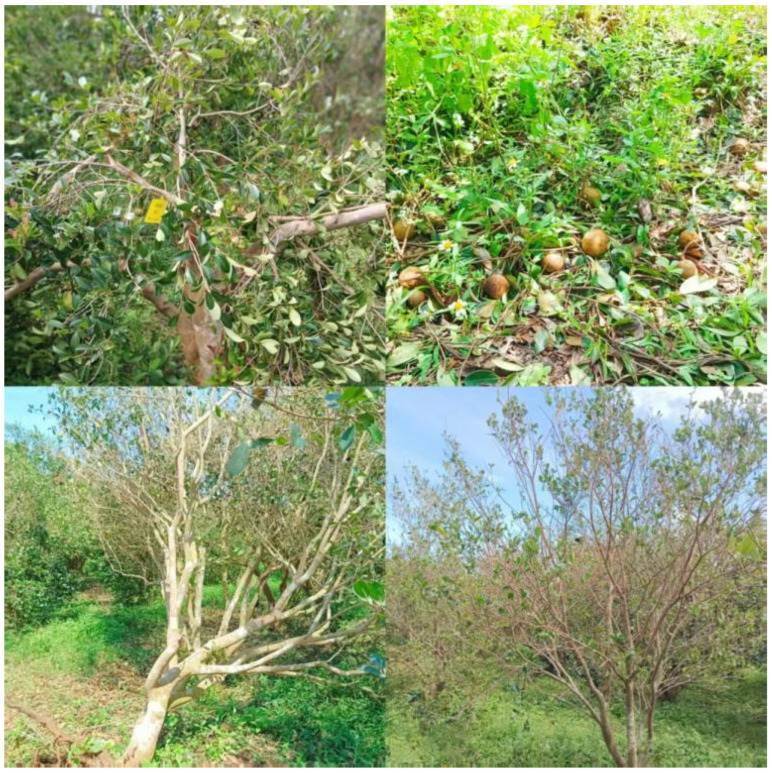
Broken branches and fallen fruits of *C. vietnamensis* trees. Note: Forest of *C. vietnamensis* in Chang’an Village, Fushan Town of the county, which was operated by Hainan Houchen Biotechnology Limited Company, 10 September 2024.

Chengmai County, Hainan Province, China, was one of the hardest-hit areas by typhoon “Capricorn” that struck on 6 September 2024. A 21-year-old forest of *C. vietnamensis* in Chang’an Village, Fushan Town of the county, which was operated by Hainan Houchen Biotechnology Limited Company, was selected as the disaster investigation plot. The information on the main environmental factors was collected from the official website of the Chengmai County People’s Government (https://chengmai.hainan.gov.cn/, accessed on 18 December 2025) are shown in Table 7. After the typhoons, trees collapsed, and some branches were broken, and a large number of leaves and fruits fell from the oil-tea camellia trees in the forest, causing severe damage (Figure 5).

The oil-tea camellia fruits need to be peeled off, followed by cleaning and disinfection [10], and then the fallen fruits are usually collected after typhoons when the soil has dried. On 10 September 2024, four days after typhoon “Capricorn,” when the ground had dried, the workers of Hainan Houchen Biotechnology Limited Company collected all the fallen fruits caused by the typhoon. Then, 30 kg of typhoon-damaged fallen fruits (HCA) were collected from these fallen fruits, which fell off 50 days before the normal harvest. On 25 October, 30 kg of normally harvested fruits (HCB) were collected from the whole oil-tea camellia forests of Hainan Houchen Biotechnology Limited Company. Taking 2 kg of randomly selected fruits as a plot, both HCA and HCB were divided into 15 replicates, respectively, for a comparative study of the two samples.

### 3.2. Treatment Methods

#### 3.2.1. Treatment of *C. vietnamensis* Seeds

The fruits were air-dried until the peels cracked, then the seeds were removed and weighed in each experimental plot. The fresh fruit seed yield rate of each plot was calculated. The fresh seeds were fixed in an oven at 105 °C for 15 min, then dried at 65 °C to constant weight, and the dry seed weight was determined. The kernel was removed, an electronic scale was used to weigh the dry kernel weight, and the dry seed kernel yield rate was calculated. After removing the seed coat, oil-tea camellia seed oil was extracted from the dry kernels by pressing with an RG-311 oil press (Dongguan Xiangju Intelligence Co., Ltd., Dongguan, China. The temperature was 200 °C). They were weighed with a 1/100 balance, and the oil yield rate of dry kernels was calculated. Finally converted to the oil yield rate of dry seeds, fresh seeds, and fruits [10].

#### 3.2.2. Physical and Chemical Properties of *C. vietnamensis* Seed Oil

The determination of peroxide value was carried out according to GB 5009.227-2016, “The Determination of Peroxide Value in Animal and Vegetable Oils” [11]. The determination of iodine value was performed under GB/T 5532-2022, “The Determination of Iodine Value in Animal and Vegetable Oils” [12]. The determination of acid value referred to GB 5009.229-2025, “the determination of Acid Value and Acidity in Animal and Vegetable Oils” [13]. The determination of saponification value was conducted following GB/T 5534-2024, “The determination of Saponification Value in Animal and Vegetable Oils” [14]. Each replicate sample was mixed.

Fresh seed yield rate of fruit (%) = A1/A2 × 100

Dry kernel yield rate of dry seed (%) = A3/A4 × 100

Oil yield rate of dry seed (%) = (A3 − A5)/A4 × 100

Oil yield rate of dry kernel (%) = (A3 − A5)/A3 × 100

Oil content rate of fruit (%) = (A3 − A5)/A2 × 100

A1: Fresh seed weight in each experimental plot.

A2: Fruit weight in each experimental plot.

A3: Kernel weight in each experimental plot.

A4: Dry seed weight in each experimental plot.

A5: Oil residue weight in each experimental plot.

#### 3.2.3. Analysis of the Composition and Content of Fatty Acids in *C. vietnamensis* Seed Oil

Fatty acid methyl esters were prepared according to the requirements of GB/T 17376-2008, “the determination of fatty acids in food” [15]. A total of 60 µL of oil sample was added to a 5 mL stoppered test tube, followed by 4 mL of isooctane to dissolve the oil thoroughly. Subsequently, 200 µL of KOH methanol solution was added, and the tube was sealed with a glass stopper and shaken vigorously for 30 s to initiate the methyl esterification reaction. The mixture was allowed to stand until fully clarified, after which 1 g of NaHSO_4_ was added, and the mixture was shaken vigorously to neutralize the excess KOH. After complete precipitation of the salts, the upper organic phase was collected and filtered through a membrane filter. The filtrate was transferred into a gas chromatography (GC) vial for subsequent analysis.

For gas chromatography-mass spectrometry (GC-MS) detection, a mixed standard of 37 fatty acid methyl esters from Shanghai Yuanye Bio-Technology Co., Ltd., Shanghai, China (No. B25881) was used for qualitative and quantitative calibration. The separation was performed on a capillary column with specifications of 30 m × 0.25 mm × 0.25 µm. The GC conditions were set as follows: inlet temperature at 220 °C, and flame ionization detector (FID) temperature maintained at 260 °C. The MS detection was conducted in total ion current (TIC) mode with a mass scan range of 50–600 *m*/*z*. A split injection mode was adopted, with a split ratio of 20:1 and a split flow rate of 24 mL/min. The column temperature program was optimized as follows: initial temperature held at 220 °C for 1 min, cooled down to 180 °C at a rate of 6 °C/min and held for 3 min, then heated up to 220 °C at a rate of 7 °C/min and maintained for 28 min. Finally, the types and contents of fatty acids in the sample were determined via GC-MS analysis. The types and contents of fatty acids were determined by the normalization method [4].

#### 3.2.4. Total Antioxidant Activity of *C. vietnamensis* Seed Oil

DPPH⋅scavenging activity, ABTS+⋅inhibitory activity, and the total reductive activity were measured using the kits such as the Total Antioxidant Capacity (DPPH⋅method) kit (Suzhou Keming Biotechnology Co., Ltd., Suzhou, China), the Total Antioxidant Capacity (ABTS+⋅method) kit (Suzhou Keming Biotechnology Co., Ltd.) and the Total Antioxidant Capacity (FRAP method) kit (Suzhou Keming Biotechnology Co., Ltd.) respectively. The experiment operation and the data analysis methods in detail can be found in the reference [4].

#### 3.2.5. Content of Bioactive Compounds in *C. vietnamensis* Seed Oil

The oil-tea camellia seed oil test sample’s volume was 7 mL, and it was added to a 20 mL headspace vial sealed immediately. The vial was equilibrated in a water bath with a constant temperature of 80 °C for 30 min, followed by headspace extraction using an aged (or desorbed) solid-phase microextraction (SPME) fiber for 30 min. Subsequently, the SPME fiber was entered into the process of GC-MS, then the method of GC-MS was detailed, and the calculations of the relative content of each volatile component can be found in the reference [4].

### 3.3. Data Analysis

Use SAS software 9.4 to perform the statistical data analysis. After selecting the univariate procedure to test the normality of the data and the glm procedure with the method of hovtest = bartlett to test the homogeneity of variance, select the *t*-test procedure to analyze the significance difference between HCA and HCB, and the REG procedure to analyze the univariate linear regression.

## 4. Discussion

### 4.1. The Cause of the Yield Loss After Typhoon Disasters

This study showed that typhoon-damaged fallen fruits led to extremely significant yield reduction, which was attributed to insufficient oil accumulation in *C. vietnamensis* fruits at this stage. Typhoon “Capricorn” happened on September 6, and this time was the period for the increase in dry weight, oil content, kernel rate, and oil rate of dry seeds or dry kernel, so not only the dry matter of the kernel was increasing, but also the oil was synthesizing [16]; typhoon-damaged fallen fruits missed the following longer critical oil accumulation period. In general, the most damage to the oil-tea camellia industry from typhoons was the huge loss of the seed oil, which also indicated the necessity and importance of typhoon disaster prevention.

### 4.2. The Contradiction Between the Peroxide Value and the Antioxidant Activity of the Seed Oil from HCA

From the results, the peroxide value of the oil from HCA was significantly higher than that from HCB; meanwhile, the antioxidant activities of the oil from HCA were significantly poorer than those of HCB. It seemed there was a contradiction. However, the peroxide value is a chemical property aiming at the fatty acids of the oil [11], yet the antioxidant activities are a type of bioactivity aiming at the bioactive compositions of the oil [4], so both actually reflect two types of quality characteristics. Analyzing it further combining with the results of the ratios of (MUFA + PUFA)/SFA, the acid value determination and the bioactive compounds identification, it could be seen that the causal relationships of these two groups are quite obvious [4,13,17]; it was true that the oil from HCA could be peroxided more easily because of its higher contents of MUFA + PUFA and was more antioxidant because of its more antioxidant compounds.

### 4.3. The Changes in the Composition of the Fatty Acids and Physical and Chemical Properties of Seed Oil After Typhoon Disasters

Although the physicochemical properties, such as peroxide value and iodine value, of *C. vietnamensis* seed oil from typhoon-damaged fallen fruits met the quality standards for first-grade products specified in oil-tea camellia seed oil (GB/T 11765-2018) [18], they were still inferior to those from normally harvested fruits. In particular, the acid value of *C. vietnamensis* seed oil from typhoon-damaged fallen fruits was far higher than the threshold for third-grade oil. The excessively high acid value in *C. vietnamensis* seed oil from typhoon-damaged fallen fruits might be due to active metabolism during the immature fruit stage, incomplete oil synthesis, and accumulation of more MUFA and PUFA, so the results of the difference in the ratios of MUFA/SFA, PUFA/SFA, and (MUFA + PUFA)/SFA agreed with the former report [19]. The higher accumulation of MUFA and PUFA led to the oil being oxidized more easily [17], and the results of the difference in the peroxide values have also confirmed it. It led to an increase in acid value and aggravated the rancidity of oil-tea camellia seed oil from the fallen fruits caused by the typhoons [20]. So, it indicated that the storage resistance of *C. vietnamensis* seed oil from the fallen fruits caused by the typhoons was from bad to worse; meanwhile, it further indicated the necessity and importance of typhoon disaster prevention.

### 4.4. The Changes in the Composition of the Bioactive Compounds and the Antioxidant Activities of Seed Oil After Typhoon Disasters

The impact of free radicals on human health is complex and dualistic. Under normal conditions, appropriate amounts of free radicals participate in human metabolic processes, such as regulating cell signal transduction, enhancing immune function, and killing foreign microorganisms, playing an important role in maintaining vital activities and health [21]. However, when there are excessive free radicals in the body or an imbalance in the antioxidant system, oxidative stress is triggered, leading to a series of negative effects such as cell damage, gene mutation, protein and lipid peroxidation, thereby inducing various diseases, including cancer, cardiovascular diseases, neurodegenerative diseases, diabetes, aging, and decreased immune system function [22]. This study showed that *C. vietnamensis* seed oil has strong antioxidant activity against different reactive oxygen species free radicals in vitro. Meanwhile, there are significant differences in the antioxidant activities between *C. vietnamensis* seed oil from typhoon-damaged fallen fruits and that from normally harvested fruits; *C. vietnamensis* seed oil from typhoon-damaged fallen fruits enhanced the antioxidant activities in vitro. Although the determination of antioxidant activity in vitro might make a huge difference from that in vivo, in a way, it still provided a new insight for typhoon disaster reduction because the healthcare quality of this product seemed to be improved.

The differences in antioxidant activity of *C. vietnamensis* seed oil might be related to the compositional variations in its bioactive components. Most compounds detected by GC-MS exhibit antioxidant properties. For example, “1,2-Benzenedicarboxylic acid, bis(2-methylpropyl) ester” showed high binding energy (5.4–5.7 kcal/mol) with pro-apoptotic proteins (such as Bax, caspase-3, and p53) in molecular docking, potentially influencing oxidative stress responses indirectly by regulating the cell apoptosis pathway [23]. Oxalic acid was a natural antioxidant that could delay the ripening of fruits like mangoes and peaches, inhibit ethylene production, and reduce reactive oxygen species (ROS) accumulation by enhancing the activity of antioxidant enzymes (such as SOD and CAT) [24]. Since ester compounds can enhance their antioxidant effects in the oil phase by improving lipid solubility [25], and oxalic acid has antioxidant properties, it could be inferred that oxalate esters retain partial antioxidant activity. Additionally, fatty acid esters such as Hexadecanoic acid, ethyl ester, and Ethyl Oleate have been reported to possess antioxidant activity. For instance, in corn silk extract, Hexadecanoic acid, as one of the main volatile components, significantly enhanced the overall antioxidant capacity [26]. Squalene could improve the activity of glutathione peroxidase (GPx), superoxide dismutase (SOD), and catalase (CAT), improving the intracellular antioxidant defense capacity (Elwekeel et al. 2023). In Salsola plants, 9-Octadecenoic acid (E)- has been confirmed to exert antioxidant effects by inhibiting free radical generation and oxidative stress responses [27]. As shown in Table 7, these compounds with antioxidant properties showed varying degrees of reduction after typhoons, even leading to the disappearance of certain compounds. This might explain why the DPPH^·^ free radicals scavenging, ABTS^·+^ free radicals inhibitory capacity, and ferric ion-reducing activity by FRAP assay of *C. vietnamensis* seed oil from typhoon-damaged fallen fruits were stronger than those from normally harvested fruits.

## 5. Conclusions

Typhoons damage to the oil-tea camellia industry not only significantly reduces the oil yield of the fallen *C. vietnamensis* fruits caused by typhoons, but also increases the acid value and peroxide value of the seed oil, which leads to going rancid and oxidizing more easily, thus making it less suitable for storage. The nutritional quality and the fatty acid content of *C. vietnamensis* seed oil from the fallen fruits caused by typhoons and normally harvested fruits met first-class standards (GB/T 11765-2018), although the seed oil from the fallen fruits caused by typhoons showed an optimizing trend in terms of fatty acid composition, so this advantage is not obvious. The seed oil from the fallen fruits caused by typhoons showed an optimizing trend in antioxidant activities, which still needs further confirmation in vivo. In general, this paper has evaluated the huge yield loss and indicated the detailed influences on the quality and the antioxidant activity of *C. vietnamensis* seed oil from the fallen fruits caused by typhoons, and has provided a new insight for typhoon disaster reduction in Hainan province’s *C. vietnamensis* production to some extent. On the other hand, the results of this paper are limited to the oil-tea camellia seed oil from the hot press process because the high-temperature extraction may alter thermolabile compounds; it is different from the other oils obtained from other processes, such as cold press or chemical extraction.

## Figures and Tables

**Table 1:** Yield traits of *C. vietnamensis* seed oil.

	Fresh Seed Yield Rate of Fruits (%)	Dry Kernel Yield Rate of Fruits (%)	Oil Yield Rate of Dry Kernel (%)	Oil Yield Rate of Dry Seeds (%)	Oil Content Rate of Fruits (%)
HCA	49.36 ± 5.76	27.07 ± 3.31	22.35 ± 7.12	4.96 ± 1.97	1.96 ± 0.78
HCB	51.20 ± 4.35 **	45.93 ± 2.02 **	43.13 ± 4.33 **	19.32 ± 1.96 **	7.33 ± 2.29 **

Note: HCA symbolizes the typhoon-damaged fallen fruits, and HCB symbolizes the normally harvested fruits. The “**” indicates a highly significant difference (*p* < 0.01).

**Table 2:** Comparison of the physicochemical properties of *C. vietnamensis* seed oil.

	Peroxide Value (%)	Iodine Value (%)	Acid Value (mg/g)	Saponification Value (mg/g)
HCA	0.07 ± 0.03 *	97.24 ± 10.48	4.72 ± 1.40 **	202.60 ± 6.47
HCB	0.05 ± 0.02	93.38 ± 9.31	1.53 ± 0.38	213.20 ± 11.89 **

Note: HCA symbolizes the typhoon-damaged fallen fruits, and HCB symbolizes the normally harvested fruits. The “**” indicates an extremely significant difference (*p* < 0.01), and the “*” indicates a significant difference (*p* < 0.05).

**Table 3:** Comparison of fatty acid contents of *C. vietnamensis* seed oil.

Fatty Acid		Relative Content (%)	
		HCA	HCB
saturated (SFA)	methyl myristate (C14:0)	0.05 ± 0.007 **	0.04 ± 0.02
	methyl palmitate (C16:0)	10.99 ± 0.24	11.09 ± 0.35
	methyl stearate (C18:0)	2.36 ± 0.097	2.51 ± 0.07 **
	methyl arachidate (C20:0)	0.05 ± 0.02	0.04 ± 0.02
	methyl behenate (C22:0)	0.007 ± 0.01	0.008 ± 0.01
	methyl lignocerate (C24:0)	0.05 ± 0.05	0.02 ± 0.05
monounsaturated (MUFA)	methyl palmitoleate (C16:1)	0.08 ± 0.006 **	0.07 ± 0.004
	methyl oleate (C18:1)	75.57 ± 0.95 **	75.13 ± 1.06
	methyl 11-eicosenoate (C20:1)	0.45 ± 0.12	0.51 ± 0.03
	methyl erucate (C22:1)	0.40 ± 0.09	0.41 ± 0.17
	methyl nervonate (C24:1)	1.05 ± 0.28	1.59 ± 0.72 *
polyunsaturated (PUFA)	methyl linoleate (C18:2)	8.56 ± 0.63	8.32 ± 0.60
	α-methyl linolenate (C18:3)	0.34 ± 0.12 **	0.23 ± 0.01

Note: HCA symbolizes the typhoon-damaged fallen fruits, and HCB symbolizes the normally harvested fruits. The “**” indicates an extremely significant difference (*p* < 0.01), and the “*” indicates a significant difference (*p* < 0.05).

**Table 4:** Equation of unitary linear regression and EC_50_ value of scavenging DPPH^·^ in *C. vietnamensis* seed oil from HCA and HCB.

Sample	Regression Equation	EC_50_ (mg/mL)	*r* ^2^
HCA	y = 0.91x + 22.638	30.09	0.9834
HCB	y = 1.29x + 3.49	36.12	0.9621
VC	y = 1.81x − 12.185	0.034	0.9475

Note: HCA symbolizes the typhoon-damaged fallen fruits, and HCB symbolizes the normally harvested fruits.

**Table 5:** Equation of unitary linear regression and IC_50_ value of inhibitory ABTS^·+^ in *C. vietnamensis* seed oil from HCA and HCB.

Sample	Regression Equation	IC_50_ (mg/mL)	*r* ^2^
HCA	y = 1.17x + 5.3095	38.31	0.9881
HCB	y = 0.89x + 5.7044	49.73	0.954
VC	y = 1.85x − 1.6492	27.9	0.9994

Note: HCA symbolizes the typhoon-damaged fallen fruits, and HCB symbolizes the normally harvested fruits.

**Table 6:** Comparison of the volatile content of *C. vietnamensis* seed oil.

	Sample	Compound	HCA (%)	HCB (%)
1	Ketone	1,5-Heptadien-4-one, 3,3,6-trimethyl-	26.08	47.76
2	Alcohols	Cyclopentanol, 1-methyl-	0.65	1.39
3		Cyclopentanol, 3-methyl-	-	0.36
4	Organic Peroxides	Hydroperoxide, 1-methylpentyl	4.90	8.25
5		Hydroperoxide, 1-ethylbutyl	3.42	5.89
6	Esters	Oxalic acid, cyclohexyl butyl ester	1.52	-
7		Oxalic acid, cyclohexyl octyl ester	0.97	1.74
8		1,2-Benzenedicarboxylic acid, bis(2-methylpropyl) ester	18.90	3.23
9		Dibutyl phthalate	25.37	3.68
10		Hexadecanoic acid, ethyl ester	0.72	-
11		11-Octadecenoic acid, methyl ester	0.06	-
12		Oxalic acid, cyclohexyl dodecyl ester	1.32	0.66
13		1,2-Benzenedicarboxylic acid, butyl 2-methylpropyl ester	-	2.77
14		Glycidyl oleate	0.10	0.14
15		l-Leucine, N-(2-chloroethoxycarbonyl)-N-methyl-, tetradecyl ester	-	3.53
16		4-tert-Octylphenol, TMS derivative	-	0.41
17		Linoleic acid ethyl ester	0.15	-
18		Ethyl Oleate	0.69	-
19	Terpenes	Naphthalene, 2,3,5,6,7,8,8a-octahydro-1,8a-dimethyl-7-(1-methylethenyl)-, 1R-(1.alpha.,7.beta.,8a.alpha.)]-	0.03	-
20	Alkanes	Squalene	4.74	2.30
21		Hexadecane	0.21	-
22		Cyclooctasiloxane, hexadecamethyl-	0.66	0.16
23		Cyclononasiloxane, octadecamethyl-	1.84	-
24		Octasiloxane, 1,1,3,3,5,5,7,7,9,9,11,11,13,13,15,15-hexadecamethyl-	0.60	-
25		Cyclohexasiloxane, dodecamethyl-	0.25	0.13
26		9-Octadecenoic acid, (E)-	3.54	-
27		Heptasiloxane, hexadecamethyl-	2.02	-
28	Olefins	1-Butene, 2,3,3-trimethyl-	-	2.72
29	Phenol	4-Methyl-2,4-bis(p-hydroxyphenyl)pent-1-ene, 2TMS derivative	-	4.94
30	Other Compounds	2-Chloroaniline-5-sulfonic acid	-	0.99
31		Indolizine, 2-(4-methylphenyl)-	-	8.96
32		Oleic anhydride	0.06	-
33		Benzimidazo [2,1-a]isoquinoline	0.68	-
34		6-Methoxy-3-methyl-2-benzofurancarboxylic acid	0.45	-

Note: HCA symbolizes the typhoon-damaged fallen fruits, and HCB symbolizes the normally harvested fruits.

**Table 7:** The key environmental factors of producing areas.

Producing Region	Elevation (m)	Sample Tree Site	Agrotype	Annual Temperature (°C)	Annual Rainfall (mm)	Annual Sunshine (h)
Changan Village, Fushan Township, Chengmai County (HC)	21	19°54′00″ N 109°54′00″ E	Red-yellow soil	24.7	2250	1900

## Data Availability

The original contributions presented in this study are included in the article. Further inquiries can be directed to the corresponding author.

## Abbreviations

HCA	Seed oil from fallen fruits caused by typhoons
HCB	Seed oil from normally harvested fruits
SFA	Saturated fatty acids
MUFA	Monounsaturated fatty acids
PUFA	Polyunsaturated fatty acids
DPPH^·^	1,1-Diphenyl-2-picrylhydrazyl radical
ABTS^·+^	2,2′-Azino-bis(3-ethylbenzothiazoline-6-sulfonic acid) diammonium salt
FRAP	Ferric ion reducing antioxidant power
CW	Clean water
Vc	Ascorbic acid
PCA	Principal components analysis
GC-MS	Gas chromatography-mass spectrometry
FID	Flame ionization detector
TIC	Total ion current
SPME	Solid-phase microextraction
